# Inhalation of Immuno-Therapeutics/-Prophylactics to Fight Respiratory Tract Infections: An Appropriate Drug at the Right Place!

**DOI:** 10.3389/fimmu.2019.02760

**Published:** 2019-11-29

**Authors:** Thomas Sécher, Alexie Mayor, Nathalie Heuzé-Vourc'h

**Affiliations:** ^1^INSERM U1100, Centre d'Etude des Pathologies Respiratoires, Tours, France; ^2^Centre d'Etude des Pathologies Respiratoires, Université de Tours, Tours, France

**Keywords:** respiratory infection, biopharmaceutics, immune-pharmaceutics, topical delivery, inhalation

## Introduction

Respiratory tract infections (RTIs) are the third leading cause of morbidity and mortality worldwide, accounting for ~4.25 million deaths in 2010, in either children, adults or the elderlies. RTIs encompass acute infections of the upper (rhinosinusitis, …) and lower airways (pneumonia, bronchiolitis, …) and are also inherently associated with chronic diseases such as chronic obstructive pulmonary disease (COPD) and cystic fibrosis (CF). In addition to premature mortality, RTIs result in a huge burden on the society considering quality-adjusted life year loss and additional pressure on the overwhelmed healthcare systems, thereby representing a major public health issue.

Antimicrobial chemotherapies (e.g., antibiotics, antivirals) are the standard interventions to prevent and to treat respiratory infections. However, their effectiveness is declining due to increased pathogen resistance, urging alternative or complementary strategies to reinforce the anti-infectious arsenal to fight RTIs. Among those under evaluation, immunomodulatory agents (immunopharmaceutics) like therapeutic antibodies (Ab) or other therapeutic proteins and vaccines may offer novel opportunities for the prevention and treatment of RTIs, by targeting pathogens and boosting the host immune system. When used in a preventive way in patients at risk, or therapeutically to stop or to limit the spread of infection, both immunopropylactics and immunotherapeutics are administered through parenteral routes (including intravenous, subcutaneous, and intramuscular) (Table 1). As demonstrated in preclinical studies, parenteral delivery may not be optimal for large molecular weight entities to treat respiratory diseases (1, 2) since they poorly reach the lung compartment. In contrast, inhalation, comprising the intranasal and oral respiratory routes, targets drugs into the respiratory tract. Currently, inhalation is used both for locally- and systemically-acting drugs as it allows a straight delivery to the diseased organ and a portal to the blood circulation, considering the extensive alveolus-capillary interface. By providing a better therapeutic index, inhalation is the gold standard for small molecules, delivered topically as an aerosol, like corticosteroids/steroids, decongestants or bronchodilators for the treatment of asthma, rhinosinusitis or COPD. Besides, it is also indicated for antibiotics (nasal and oral inhalation), a local-acting protein therapeutic—Dornase alpha (Pulmozyme®, oral inhalation), a mucolytic agent for patients with CF and an influenza live vaccine (FluMist® Quadrivalent, nasal inhalation).

**Table 1:** Marketed immunotherapeutics and immunoprophylactics for infectious diseases.

**Target**	**Product**	**Category**	**Sponsors**	**Administration route**	**Date of approval**	**Indication**
RSV	Synagis	Monoclonal antibody	MedImmune	IM	1998	Prophylaxis
Influenza	Afluria	Inactivated vaccine Quadrivalent	Seqirus	IM	2007	Prophylaxis
	Fluad	Inactivated vaccine Trivalent	Seqirus	IM	2015	Prophylaxis
	Fluarix	Inactivated vaccine Quadrivalent	GSK	IM	2012	Prophylaxis
	Flublok	Recombinant vaccine Quadrivalent	Protein Sciences Corporation	IM	2013	Prophylaxis
	Flucelvax	Inactivated vaccine Quadrivalent	Seqirus	IM	2012	Prophylaxis
	Pandemic influenza vaccine H5N1	Recombinant vaccine	Medimmune	IN	2016	Prophylaxis
	FluLaval	Inactivated vaccine Quadrivalent	ID Biomedical Corporation of Quebec	IM	2013	Prophylaxis
	FluMist	Live-attenuated vaccine Quadrivalent	MedImmune	IN	2003	Prophylaxis
	Fluzone High Dose	Inactivated vaccine Quadrivalent	Sanofi Pasteur	IM	2014	Prophylaxis
	Fluzone	Inactivated vaccine Quadrivalent	Sanofi Pasteur	IM	2009	Prophylaxis
	Fluvirin	Inactivated vaccine Trivalent	Seqirus	IM	1988	Prophylaxis
Measle	Proquad	Subunit vaccine	Merck	SC	2005	Prophylaxis
	M-M-R II	Subunit vaccine	Merck	SC	2014	Prophylaxis
Smallpox	ACAM2000	Live vaccina virus	Emergent Product Development	Percutaneous	2007	Prophylaxis
*Mycobacterium tuberculosis*	BCG Vaccine	Live-attenuated vaccine	Organon	Percutaneous	2011	Prophylaxis
*Streptococcus pneumoniae*	Pneumovax 23	Subunit vaccine	Merck&Co	IM	1983	Prophylaxis
	Prevenar 13	Subunit vaccine	Wyeth Pharmaceuticals	IM	2010	Prophylaxis
*Bordetella pertussis*	Daptacel	Subunit vaccine	Sanofi Pasteur	IM	2008	Prophylaxis
	Pediarix	Subunit vaccine	GSK	IM	2002	Prophylaxis
	Kinrix	Subunit vaccine	GSK	IM	2008	Prophylaxis
	Quadracel	Subunit vaccine	Sanofi Pasteur	IM	2015	Prophylaxis
	Pentacel	Subunit vaccine	Sanofi Pasteur	IM	2008	Prophylaxis
*Haemophilus influenzae*	Hiberix	Subunit vaccine	GSK	IM	2009	Prophylaxis
	ActHIB	Subunit vaccine	Sanofi Pasteur	IM	1993	Prophylaxis
	PedvaxHIB	Subunit vaccine	Merck	IM	1989	Prophylaxis
*Bordetella pertussis* *Haemophilus influenzae*	Infanrix	Subunit vaccine	GSK	IM	1997	Prophylaxis
	Vaxelis	Subunit vaccine	MCM Vaccine	IM	2018	Prophylaxis
*Bacillus anthracis*	Anthim	Monoclonal antibody	Elusys Therapeutics	IV	2016	Prophylaxis/Therapy
	Abthrax	Monoclonal antibody	GSK	IV	2012	Prophylaxis/Therapy
	Biothrax	Subunit vaccine	Emergent BioSolutions	IM	2016	Prophylaxis

*IM, intramuscular; IN, inhalation (nasal); SC, subcutaneous*.

## Local-Acting Immunopharmaceutics Delivered by Inhalation

There are accumulating evidences that administration of anti-infectious Abs, protein therapeutics (e.g., cytokines) and vaccines, to the upper and/or lower respiratory tract by inhalation, with the purpose of inducing a local action, is effective (3). Several preclinical studies showed the superiority of immunopharmaceutics administered topically to the respiratory tract in RTI models, in both therapeutic and prophylactic regimens. For instance, inhalation of anti-infectious Abs in models of pneumonia using *Pseudomonas aeruginosa* or influenza virus conferred higher protection and greater therapeutic response, respectively, compared to parenteral route administration (4, 5). Besides, other immunoprophylactics delivered through the respiratory route such as immunocytokines (e.g., IL-7 Fc) (6) and live-attenuated vaccines (7) showed superior performances over conventional routes against airborne viruses, in mice and non-human primates, respectively. Conversely, restricting the response to the site of action for pleiotropic molecules (e.g., IL-7 Fc), envisioned as adjuvant molecule, may reduce systemic side-effects. As reported for anti-infectious Abs, the inhaled route may also enable a higher efficacy with a lower dose (4). This means that the inhaled route may allow, in the future, to alleviate the financial burden of immunopharmaceutics (in particular Abs), which may exceed the ability of both individual patients and the healthcare systems to sustain them. Additional benefit of the inhaled route includes its non-invasiveness, offering a better comfort for patients, in particular those with chronic respiratory infections, and thus preventing additional healthcare costs. Besides, needle-free vaccination may prevent the risk of cross-contamination and facilitate mass vaccination efforts.

**Figure 1:**
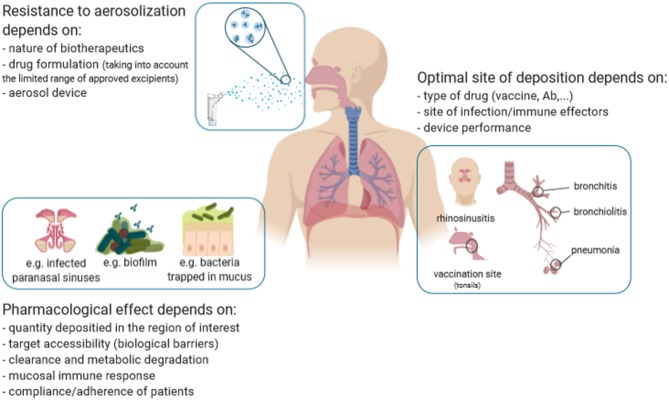
The multifaceted features from the development of inhaled immunopharmaceutics.

However, beyond clear preclinical proofs of concept and obvious theoretical advantages of the inhalation route for immunotherapeutics and -prophylactics, few of these benefits have materialized in the clinic (Table 1). Except for Flumist® Quadrivalent (Astrazeneca), an intranasal live attenuated influenza vaccine, other marketed immunoprophylactics vaccines (including those against *Streptococcus pneumoniae, Haemophilus influenza, Mycobacterium tuberculosis, Bordetella pertussis* or measles and Ab (anti-RSV Pavilizumab)—are administered systemically. Similarly, none of the protein therapeutics is given by inhalation. Recently, Ablynx developed an inhaled anti-RSV trimeric nanobody® (ALX-0171) for therapeutic purposes. Despite promising results in several animal models, the development has been interrupted due to insufficient evidences of efficacy during Phase 2 trial in children (in Japan). In 2019, only one phase 2 trial with an inhaled anti-infectious protein therapeutics is still ongoing (NCT03570359) assessing the efficacy of topical lung delivery of IFN-β1a (SNG001, Synairgen/Astrazeneca), as an immunostimulant to treat COPD exacerbations. Overall, this highlights the complexity of developing inhaled biopharmaceuticals and points out the persisting hurdles (Figure 1).

## Challenges for the Development of Inhaled Immune-Therapeutics/Prophylactics

The instability of immunopharmaceutics and vaccines often emerges as a challenge for inhalation delivery. Therapeutic proteins and vaccines are sensitive to various conditions which may alter their structure, thereby decrease their activity. Delivering a drug through the inhalation route implies either spraying, drying or aerosolizing, which is associated with multiple stresses (shearing, temperature, air/liquid interface, …) potentially deleterious as widely discussed elsewhere (8, 9). To deal with this, both the device used for the generation of the aerosol and the formulation must be adapted, as successfully reported for Ab-based therapeutics (3, 10). However, the excipients must be adapted for respiratory delivery. The choice of mucosal-licensed adjuvants, which should be exempt of intrinsic immune-toxicity, and the instability of the associated carrier [e.g., nanoparticles, liposomes, immune stimulating complexes (ISCOMs)] is particularly challenging for the inhalation delivery of vaccines, especially those of the latest generation (e.g., T, B-epitope-based vaccines). The drug and device combination yields proper aerodynamical properties (particle size, flow rate, …) to achieve the anticipated deposition in the appropriate area of the respiratory tract. Indeed, appropriate deposition to the anatomical site is mandatory to ensure an optimal efficacy. On one hand, this depends on the drug formulation (e.g., surface tension and viscosity for liquid formulation) (11) and device performances to allow the therapeutic agent to reach the site of infection (Figure 1), by this means the microbe. For lung infections, most pneumonia consists of an aggregate of trachea-bronchitis and alveolar infections. Theoretically, this clinical condition may benefit from a uniform distribution all over the lungs, with a polydisperse aerosol (ranging 1–5 μm). However, several pathogens are associated with specific anatomic localization, like *S. pneumoniae*, which is mainly found in the alveolar spaces, thereby requiring low-size aerosols (<2–3 μm) to be targeted. On the other hand, delivery to the mucosal-associated lymphoid tissue (MALT), located in the tonsils, would be more adapted for vaccines to induce an adaptive immune response, since MALT plays a central role in the primary respiratory immune defense (Figure 1).

Biological barriers are additional hurdles to overcome and apply to all inhaled anti-infectious agents (12). First, a pathogen can “hide” itself inside host cells like *M. tuberculosis* in alveolar macrophages, thus being more difficult to be targeted by immunopharmaceutics. Other pathogens may produce extracellular barriers like the biofilm matrix produced by *P. aeruginosa* in the context of chronic lung infections. This biofilm acts as a diffusion barrier, preventing inhaled immunopharmaceutics from reaching their molecular target. Antibody-based fragments, such as fragment antigen-binding (Fab) and single-chain variable fragments (scFv) might be more efficient in crossing over the biofilm, like they penetrate better solid tumors (13), and eradicate *P. aeruginosa*. Secondly, the host physical defenses, which prevent foreign particles from penetrating into the respiratory tract, may limit the accessibility of inhaled immunopharmaceutics to their target. Among them, the mucus and the mucociliary escalator are highly efficient clearance mechanisms (14, 15). The development of mucoadhesive formulations may be helpful to enhance the bioavailability of inhaled drugs (16). In contrast, anti-adhesive molecules, such as polyethylene glycol may facilitate immunopharmaceutics translocation through the mucus blanket, as shown *in vitro* (17) and *in vivo* (18) for other applications. It is noteworthy that, in some pathological conditions (e.g., chronic sinusitis, CF and COPD), the mucus gets thicker. In CF, the mucus exhibited an increased density of disulfide cross-links, further tightening the mucus mesh space, thereby reinforcing its steric barrier potency to immunopharmaceutics (19). To date, overcoming this physical barrier has not been addressed in the design of inhaled immunopharmaceutics. Other biological barriers include alveolar macrophages and the pulmonary surfactant layer in the alveolar region. While the molecular interactions between inhaled particles and the surfactant are largely unknown, some evidences indicate that surfactant proteins may facilitate the uptake of inhaled particles by alveolar macrophages (20). Alveolar macrophages patrol the airways and phagocytose inhaled organic (including pathogens) and inorganic particles ranging between 0.5 and 5μm (21). Interestingly, the size-discriminating property of their phagocytosis potency has led to the development of innovative approaches for inhaled drugs, in which carrier entrapped-particles of smaller or larger size are inhaled to escape the alveolar macrophage phagocytosis and to provide a better controlled drug release [(22, 23); Figure 1]. This strategy is investigated for mucosal vaccines to prevent the degradation or denaturation of the peptide/antigen, to sustain its release and favor delivery and adjuvancy (24).

The lung mucosa is a metabolic active environment (25). The presence of proteases [which is more prevalent in the nasal mucosa (26)] may degrade therapeutic proteins before they reach their targets. In addition to host enzymes, bacterial pathogens, like *P. aeruginosa*, release additional proteases, which may metabolize respiratory-delivered drugs (27). In this context, the presence of protease inhibitors in the formulation of inhaled protein therapeutics may improve their pharmacokinetics and efficacy, as previously demonstrated for inhaled peptides such as insulin and calcitonin (28). Furthermore, the encapsulation of protein therapeutics into liposomes may also improve stability and reduce the frequency of dosing (29). This strategy has already been clinically validated for the pulmonary delivery of antibiotics (30). Of note, respiratory diseases are often associated with an impairment of the protease/anti-protease balance. In CF, high levels of proteases are a result of the chronic infection and inflammation induced by *P. aeruginosa* (31). This proteolytic environment self-perpetuates the intensity of inflammation, induces mucus hypersecretion and respiratory tissue damage, which may ultimately affect inhaled immunotherapeutics (Figure 1).

## Conclusion

Compared to the expansion of biopharmaceutics (excluding non-recombinant vaccines) in all medical areas, the field of inhaled protein therapeutics/vaccines has stagnated, with only few drugs approved so far. Despite promising preclinical data and significant advances on macromolecule inhalation, a definitive demonstration that effective and intact inhaled immunopharmaceuticals could be delivered (topically) to humans is still lacking.

Although, we cannot rule out that the recent failures of inhaled biopharmaceutics (Exubera and ALX-0171) make it challenging, to our opinion, it may be time for thinking carefully where inhalation may have the edge over other routes: “finding the right use for this modality!” They may be many possibilities considering the unmet clinical needs for respiratory diseases and the growing market of immunopharmaceutics. But the inhalation route must be envisioned and integrated early taking into account the disease/population, the target, the drug and the device (Figure 1), rather than adapting an approved molecule for the inhalation route. RTIs are undoubtedly an appropriate clinical situation for inhalation, if we consider the importance of matching the delivery of immunoprophylatics or immunotherapeutics to their site of action. Anti-infectious macromolecules may certainly benefit from the success of inhaled antibiotics, but it is critical to remember their precise molecular nature associated with a unique pharmacokinetics profile when considering their development for inhalation. Besides, the recent report of a universal flu vaccine, comprised of Ab-based therapeutics (VHH) produced by an adeno-associated virus delivered intranasally pushed further the boundaries of the potential of the inhalation route for immunoprophylactics (32).

## Author Contributions

TS, AM, and NH-V participated in the review of research. NH-V prepared figure. TS and AM prepared table. All authors contributed to the manuscript.

### Conflict of Interest

The authors declare that the research was conducted in the absence of any commercial or financial relationships that could be construed as a potential conflict of interest.
